# Weight Matters—Factors Influencing Eating Behaviors of Vulnerable Women

**DOI:** 10.3390/nu11081809

**Published:** 2019-08-06

**Authors:** Marcela Vizcarra, Ana María Palomino, Lorena Iglesias, Alejandra Valencia, Patricia Gálvez Espinoza, Andiara Schwingel

**Affiliations:** 1 Department of Kinesiology and Community Health, College of Applied Health Sciences, University of Illinois at Urbana-Champaign, Champaign, IL 61820, USA; 2 Department of Nutrition, College of Medicine, Universidad de Chile, Santiago 8380453, Chile; 3 Department of Primary Care and Family Health, College of Medicine, Universidad de Chile, Santiago 8900085, Chile

**Keywords:** eating behaviors, women, weight status, qualitative research

## Abstract

Women from low socioeconomic backgrounds are more affected by obesity than men. The influence of weight as a determinant of women’s eating behaviors has seldom been studied, especially in Latin America. In this study, we analyzed the food choices of vulnerable women according to their weight status. We conducted photo-elicitation interviews with 31 women from low-income neighborhoods in Santiago, Chile. Weight and height were measured and participants were divided into normal weight (*n* = 9), overweight (*n* = 15), and obese groups (*n* = 7) according to World Health Organization (WHO) body mass index (BMI) categories (*p <* 0.001). Quantitative and qualitative approaches were used for the analysis. Women in overweight and obese groups described more about their families, temporality, financial issues, and food perception. When weight groups were analyzed separately, more factors explaining eating behaviors were found (mental and physical health, body dissatisfaction, gender role, and obstacles for eating healthy) in the obese group. Results suggest that women with obesity or overweight based their diets on more internal and external factors than did normal weight women. This study contributes to our understanding of why changing behaviors can be difficult in women with obesity. Health care providers should consider these factors in the implementation of programs to address the need for a healthy diet for overweight and obese women.

## 1. Introduction

Helping people to meet dietary recommendations represents an enormous challenge in the public health agenda. Unhealthy eating behaviors, such as a low fruit and vegetable consumption or a high calorie-dense food intake (e.g., fast food), have been associated with approximately 1.7 million deaths annually and with 16 million disability-adjusted life years (DALYs) [1]. In addition, diet plays an important role in nutrition-related diseases, such as obesity and diabetes [2,3]. While extensive efforts have been made to change unhealthy eating behaviors, many challenges exist when trying to change these [4] and other health behaviors [5]. Therefore, a deeper understanding of how these behaviors are formed could contribute to reducing the health problems associated with them.

Today, obesity represents one of the biggest nutrition-related problems, affecting more adult women than men worldwide (15% vs. 11%) [6]. The imbalance between the sexes is even more pronounced in Latin American and Caribbean countries where women are 10% more likely to be obese than men [7]. Furthermore, women from lower socioeconomic backgrounds have an even greater prevalence of obesity [8,9]. Chile has one of the highest prevalence rates of obesity in relation to other Organization for Economic Co-operation and Development (OECD) countries (34.4%) with obesity rates only slightly less than those found in the United States [10]. Among the lowest socioeconomic groups, the prevalence of obesity in Chile is as high as 43.2% overall, with even higher figures among women [11].

This difference in obesity rates can be attributed to poor diet among women of lower socioeconomic status (SES). Women from higher SES groups eat more fiber from whole-grain bread and grains than those from lower SES groups; women from middle and higher SES groups also eat fewer chips, savory snacks, and sweets [12]. Pechey and Monsivais found that people from higher SES groups spend more on food, which was associated with healthier purchases [13]. In addition, women from lower SES groups buy takeaway food from fast food restaurants more frequently than do women from middle and upper SES groups [14], and people from higher SES groups report less fast food consumption [15]. Ball et al. found that the intake of fruits and vegetables differs depending on a woman’s SES [16]. They also found that women from a lower SES are less likely to report health-related motives for choosing food. In Chile, according to the most recent National Food Intake Survey, people from lower SES groups ingest more calories than those from higher SES groups [17]. In addition, a study of female users of the Chilean Public Health System concluded that most of them had unhealthy diets, particularly those with the lowest incomes [18]. This evidence suggests that women of low SES in Chile, as in other parts of the world, have the highest risk of gaining weight and suffering the consequences of nutrition-related diseases and disorders.

When trying to understand eating behaviors in vulnerable populations such as women, research has revealed the influence of multiple factors. At the individual level, the literature describes variables such as personal preferences [19,20], level of education [21,22], nutritional knowledge [23,24], and psychological variables [25,26], among others. At the interpersonal level, friends and family, especially children, represent important influences on women’s diets [27,28,29,30]. Additionally, from a more environmental standpoint, access to affordable food plays an important role in women’s food-related choices [14,31,32,33]. Despite their contributions, most of these research studies have been conducted in developed countries only.

By contrast, there are few studies that have explored factors that influence eating behaviors of women from low SES in Latin America and Chile. A study from rural Mexico indicated that women selected food based on family preferences, money, time for cooking, and nutritional content of food [34]. An earlier study from Chile found that women often considered their individual needs as secondary to other factors. This study found that knowledge and time pressure were important factors influencing women´s diet in vulnerable neighborhoods [35]. This shows that more research is needed to understand the complexity of women´s food-related choices in Latin American countries such as Chile, where women from low SES are more affected by overweight and obesity.

Prior research into the eating behaviors of women from low SES groups has failed to explore the influence that a woman’s weight has on her food choices and eating habits. This is a significant shortcoming, as women may make different food-related decisions according to their weight status. A previous study conducted by Dressler and Smith found that differences exist between lean/normal weight and overweight/obese, low-income women with respect to how they explain their dietary choices [36]. Understanding how women with normal weight, overweight and obesity make their food choices could help to clarify what are the challenges and facilitators of behavior change within each of these population groups. This information will help to tailor health interventions to meet the specific needs and values of women according to their weight status. In this context, the aim of the current study was to analyze the influences of weight status on the food choices of women from lower SES groups.

## 2. Materials and Methods

### 2.1. Study Design and Ethics

This current study was part of a larger qualitative study conducted in Chile that intended to increase our understanding of the “food world” of Chilean women from low SES groups by exploring factors related to eating behaviors and food-making decision processes [30,37]. In that study, we found seven factors were the drivers of women’s food decisions: family, individual preferences, temporality, perceptions about food, financial issues, special moments, and food availability [30]. This study extends that research by focusing specifically on the impact of a woman’s weight status.

The research was conducted according to the guidelines from the Declaration of Helsinki, and all procedures involving human subjects/patients were approved by the Institutional Review Board at the University of Illinois at Urbana-Champaign (approval number 11122014) and from the University of Chile’s Ethics Committee (approval number 138-2014). Written informed consent was obtained from all subjects.

### 2.2. Participants Selection

We recruited 53 Chilean women living in Santiago, Chile. To participate in the study, they had to have at least one child younger than 12 years old and live with a husband/partner in a low socioeconomic urban neighborhood, as defined by the Chilean Social Priority Index [38]. This index considers education, income, and health variables to classify neighborhoods according to their vulnerability. In addition, participants answered a sociodemographic Supplementary Material  questionnaire to verify their low-income status.

We used a purposive sampling method [39] employing face-to-face contacts in waiting rooms at four public health care centers and two community organizations. This allowed us to reach participants from seven lower socioeconomic neighborhoods in Santiago. Two researchers coordinated the recruitment of participants, inviting women to participate through face to face interactions. The researchers used a checklist to assess inclusion criteria. If participants met the criteria, they were invited to participate in the study. We excluded women for the following reasons: they did not complete the photo-elicitation assignment, they reported not having enough time to participate in the study, or they did not respond to our attempts to schedule a research visit. Thirty-one women completed the study.

### 2.3. Data Collection

From November 2014 to March 2015, we conducted semi-structured interviews using photo-elicitation (PE), a methodology in which photos taken by participants are used during interviews to stimulate discussion between the participant and the researcher [40]. Each participant received a disposable camera with a capacity for 27 pictures and was trained to use it. After this training, participants were asked to take approximately 20 pictures of their “food world.” The decision to take 20 pictures was based on protocols adopted in previous research studies similar to ours [41]. We encouraged them to take pictures not only of meals but also of any important aspects of their lives that they considered relevant to their “food world.” Participants took pictures within a three-week period. To ensure that participants were completing their assignment, we called participants via phone during this period. After they finished their assignment, participants returned the camera, and we developed two copies of each of the pictures; one was kept by the researcher, and the second one was given to the participant.

At the start of the interviews, participants were asked to review their pictures and select five to seven that they most wanted to share or that they thought were particularly interesting. The interviewer also selected five to seven additional pictures from those that the participants did not select. All pictures taken by the participants were used in the selection process, including those that were apparently unrelated to the food world. This approach allowed us to prevent researcher bias and helped us to understand the context of the participants’ food worlds and to build rapport with the participants. Participants titled the selected photos [29] and explained why they gave them the corresponding title. Then, the interviewer used the Shaffer’s SHOWeD technique [42] to initiate the discussion. This technique includes questions such as “What do you see in this picture?”; “What is happening in this picture?”; “How does this relate to our life?”; “Why does this problem, concern, or strength exist?” and “What can we do about it?” These questions allowed participants to explain the picture in an open way. The interviewer asked follow-up questions to clarify points from the participants’ narrative. A complete detail of the methods can be found elsewhere [37]. Data saturation was reached in interview number 22 for the whole group, which meant that no new topics subsequently appeared in women´s discourses [43].

In addition to PE interviews, participants’ weight and height were measured, following a standardized protocol [44,45].

### 2.4. Data Analysis

We used both qualitative and quantitative approaches for data analysis. Weight and height were used to calculate body mass index (BMI), which allowed us to divide participants into three groups: normal weight group [NW] (BMI between 18.5 and 24.9), overweight group [OW] (BMI between 25 and 29.9), and obese group [OB] (BMI above 30), according to World Health Organization (WHO) criteria for adult populations [46].

We divided the qualitative analysis into two parts. First, we used the thematic analysis done in our previous research. Five researchers from the team analyzed 10 interviews that were randomly selected. These interviews were coded by each researcher, creating a preliminary codebook for each. Then, the researchers met regularly to discuss the analysis to generate a final codebook. The final codebook was used to codify all interviews. After this, the research team met again to discuss the categories and themes of the codebook. The team identified seven themes (family, preferences, temporality, perceptions about food, financial issues, special moments, and food availability). On that basis, we conducted a comparative analysis for each theme within each weight group by looking for different and common patterns within each theme, defining a pattern as a repeated idea within a theme [47]. Then, we developed a contrast table to analyze the differences between those patterns per group [48]. In doing this, we found that four of the seven themes from the first study behaved differently between groups. In order to explore these differences more thoroughly, we used a content analysis to determine the percentage of participants who mentioned a theme, and the number of times that each theme was mentioned during the interviews, which we called ‘repetition’ in this study. We included repetition in this analysis by looking at the intensity of each theme across weight status groups.

Normality in the variables was examined using the Shapiro Wilk test. Analysis of variance (ANOVA) or the non-parametric Kruskal-Wallis test were used to conduct comparisons of the variables between the three weight status groups; results from these tests are shown as mean ± standard deviation (SD) or median and interquartile range, respectively. Two-sided Fisher’s Exact test was used to compare categorical variables. Statistical significance was set at 0.05. Statistical analyses were conducted using SPSS v.24.

After the comparative analysis of each theme per weight status group as well as the content analysis, two researchers conducted a second thematic analysis for each group. This analysis allowed us to identify additional themes that we did not find in the entire sample of participants. We also created a contrast table to compare these new themes between groups and calculated the percentage and repetition of these themes. For this part of the analysis, we agreed that a theme was present when it was mentioned by more than 75% of the participants in each weight status group [48]. Given that the size of the group of women with obesity was smaller than all the other groups, we decided that in this group, a theme was present when it was mentioned by 71% of the group (i.e., five women).

Patterns identified from both analyses are summarized utilizing the words that the participants used to describe themselves. We included direct quotes from participants to exemplify the results. In addition, we used letters to identify participants, and we added the letters *NW* for normal weight participants, *OW* for overweight participants, and *OB* for participants with obesity. We used QSR International’s NVivo 10 Qualitative Data Analysis Software in order to organize the qualitative data and obtain information about quantitative variables.

## 3. Results

The participants from the three groups exhibited very similar sociodemographic characteristics; only BMI was statistically different among the groups (Table 1). As for themes, both similar and unique themes and patterns were found between groups. The results are presented according to the analysis described in the methods section.

### 3.1. Themes by Weight Group Status

From the seven themes found across the entire sample (family, preferences, temporality, perceptions about food, financial issues, special moments, and food availability), four were found to show different patterns among the weight status groups: family, temporality, financial issues, and perceptions about food. In Table 2, we present these differences for each group. Table 3 and Table 4 present the percentages of participants who mentioned a theme, and the number of repetitions, respectively. No statistical difference was found for any of the quantitative variables analyzed, however some tendencies will be described in the following sections.

#### 3.1.1. Family

In all three groups, “family” was consistently one of the most important influencers on participants’ food-related behaviors as it was mentioned by 100% of participants in each group. However, the OW and OB groups tended to mention it more often during the interviews than those of the NW group (Table 2). There were some common themes between the groups, but there were two themes that were found only in the OW and OB groups. The OW and the OB groups had more to say about how their families influence their “food world” than the NW group (Table 3). First, participants indicated that they cooked or bought specific types of foods due to the preferences of their family members. It was in these two groups (OW, OB) where it was more likely that participants put their families’ preferences over their own since they knew what their husbands/partners or children liked. This is exemplified in the following quotes: “In my house, they [her husband and daughters] are picky, they do not like this… they do not like that… so I get tired of that, and I try to make something that everybody likes…” (C27-OW). “…furthermore, she [her daughter] does not like eating salads… so I think: ‘Why am I going to prepare a salad [just for her],’ so I eat in a simple way [with no salads]” (D7-OB). Second, both the OW and OB groups said that they ate or cooked certain foods because it represented a family routine that they and their family enjoyed.

Moreover, in the OB group two additional themes were found that were not present in the other BMI groups. First, women in the OB group stated that they cooked some meals because they tried to keep their families healthy, indicating that they were in charge of the family’s wellbeing. For example, a woman stated, “For me, it will be ok for me if I have [just] a lettuce salad…but, what am I going to give the kids? They cannot eat just a lettuce salad because they are growing up…” (Y53-OB). Some of them also mentioned that they took greater care of their families’ health than their own health. Second, OB participants depended more on the opinions of others when they needed to think about what to cook or buy. They trusted in other female voices in the family, especially those who lived with other women in the same house, such as mothers or mothers in law.

#### 3.1.2. Temporality

Temporality was found in more than 85% of participants, and it was equally discussed among the three groups (Table 2). This theme had two dimensions: one related to the day of the week and the other related to the season of the year. In relation to what happened during weekends as compared to weekdays, participants from the three groups said they bought or cooked different foods for Saturday and/or Sunday meals. However, the OW and OB groups tended to justify these food changes on the grounds that they tried to make weekends special days for their families. For instance, an overweight woman said, “Sometimes, when my husband is here [at home during the weekends], we go to his mom’s or my mom’s house…so, I do not have to cook, we eat at another house…” (MI32-OW). Similar patterns were found in the three groups in relation to the seasons throughout the year. However, the OW and OB groups indicated that the different seasons of the year impacted their fruit and vegetable intake because a higher consumption of fruits and vegetables was reported in the summer and a lower one in the winter. They explained this change was due to the food availability during the different seasons and the outdoor temperature. For example, a participant indicated, “…during the summer you can get more fruits. In the summer, you can go to one store or another and you are going to find them [different kinds of fruits] …but during the winter…nothing… you can’t even find special fruits at the supermarket [such as melon or watermelon] …” (C13-OB).

#### 3.1.3. Financial Issues

About 90% of participants in all groups mentioned some situation related to “financial issues” (i.e., situations associated with purchasing food). A trend was observed in the OB group as they discussed this theme more frequently than the other two groups (Table 2).

**Figure 1:**
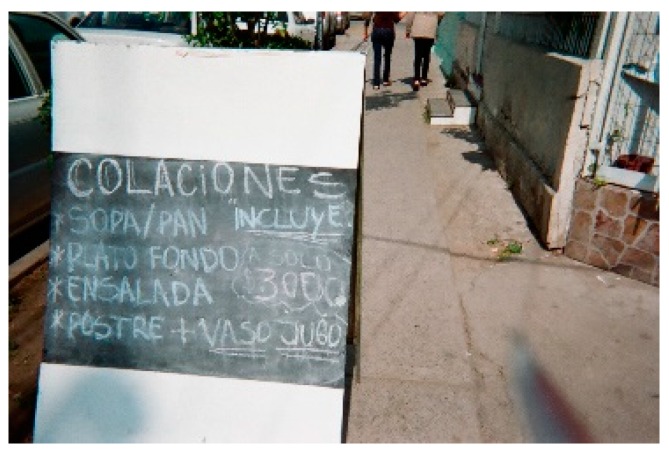
Food advertisement from a restaurant that includes different dishes in a menu and price.

Participants from each of the three groups pointed out that, in most cases, they have control over money at home. Nevertheless, only members of the NW and OW groups said that they had to refrain from eating certain foods. The participants’ avoided dining at restaurants and/or did not have the ability to buy certain kinds of meat such as fish or beef. They avoided these foods because they did not have enough money to buy them or because they preferred to spend their money on other foods that were more needed by the family. A woman with normal weight indicated, “If I pay for what appears in the picture (Figure 1) … I could not eat the next day…” (P10-NW).

The NW and OB groups also mentioned that they always looked for the cheapest foods in the market, as they tried to save money. Some of the money-saving strategies they mentioned were to: select the cheapest places to buy food, select foods that they knew were going to be enough for all family members, and look for food on sale. When money problems were mentioned by the OB and OW groups, it was usually in relation to not having enough money to buy all the food that they wanted for their families or for themselves. In turn, the fact that certain kinds of foods were bought only when participants had enough money was a pattern only found in the OW group. In that context, “having money” referred to the moment in which they or their husbands received their paycheck (in the middle or at the end of the month).

Finally, most of the participants from the OB group mentioned some foods that they perceived as expensive: certain kinds of meat; vegetables such as tomatoes and avocados; and some fruits such as strawberries and cherries. This particular point linked participants’ perceptions about whether or not they could eat healthier to their financial situation. For instance, a participant indicated, “… Healthy foods are expensive… those whole grain cookies cost 2000 [about 3 dollars], light cheese: expensive… fish: expensive… an egg is more expensive than a hamburger” (S12-OB).

Some obese participants who visited a nutritionist for advice stated that the food recommendations from these professionals were expensive. For this reason, they could not follow that kind of diet for a long period of time.

#### 3.1.4. Perceptions about Food

Different kinds of perceptions about food were mentioned by all participants, repeating this theme a similar number of times during the interview (Table 2).

Unlike the other themes, no common patterns were found in this theme among the three groups, but the NW and OW groups shared two patterns. First, both groups indicated that they perceived certain foods as healthy or unhealthy and that they would, therefore, eat or not eat those foods. These perceptions came from what they watched on television, recommendations they had received from others, and their own level of nutritional knowledge. The second shared pattern is related to the participants’ knowledge. We observed that participants from these two groups were able to talk about the nutritional content or the healthy content of different foods and that this knowledge helped them to eat higher or lower amounts of certain foods. For example, a normal weight participant said, “…because grapes have several properties… they have antioxidant and anti-inflammatory properties … [grapes] help to repair the gut, clean the blood… [they are the best for the fasting process; they prevent cancer” (B3-NW).

**Figure 2:**
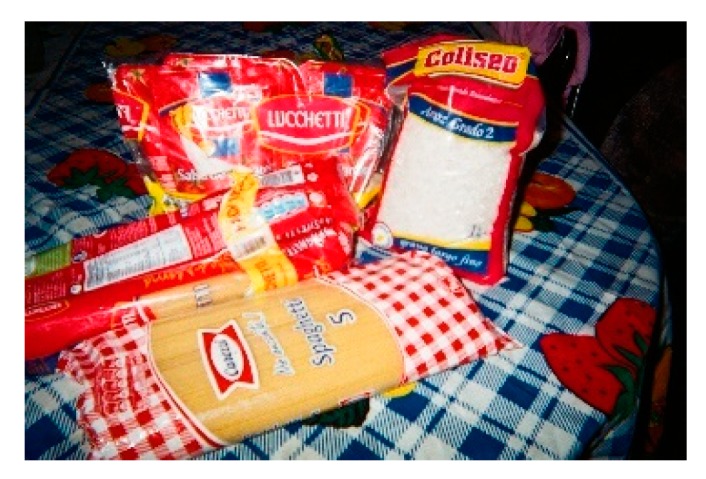
Usual food that she has in the pantry.

The OW and the OB groups also shared two patterns. Both groups indicated that there were some essential foods that they had to have at home. Participants were always worried about buying those foods because they were important for them and their families. A participant mentioned, “This [Figure 2] is what can never be missing from our pantry… they never fail: [tomatoes] sauce, rice, pasta” (Y53-OB).

In addition, OW and OB groups mentioned some foods that were related to increased body weight. It was in these two groups where the perception of different foods as “enemies” was found, and for this reason, participants tried to avoid or reduce them in their diets. The main foods that these groups tried to avoid were bread and soda.

We found a unique pattern in the OB group because they mentioned perceptions about foods that are based on beliefs and myths. Some of these were that proteins and carbohydrates must not be mixed, that milk is not necessary for the adult population, or that some infusions can burn fat. These beliefs led them to eat more or less of certain foods.

### 3.2. New Themes by Weight Status

In the analysis per group, we found five new themes. Most of them belonged to the OB group. These themes for each weight status group are shown in Table 5.

#### 3.2.1. Themes Found in the Obesity (OB) Group Only

##### Emotional and Physical Health

Five out of seven OB women mentioned issues related to emotional health in their interviews. Anxiety was the most frequently mentioned issue, as they indicated that this made them eat more, without having a reason to do so. None of these participants indicated that this anxiety had been diagnosed by a health care provider. OB participants also mentioned that it was very difficult to try to control themselves or to stop eating certain kinds of foods, and that they associated these moments with feelings of sadness. In addition, some participants defined this anxiety in terms of being hungry; for example, a participant pointed out, “I think it [anxiety] occurs because I get a pain in my stomach, like it [the stomach] is saying that ‘I am hungry… I am hungry’… I have felt this sensation at 1am… I try to eat less, but the problem is that I get anxious, and instead of not eating, I eat… eat and eat…” (B52-OB).

Most OB participants also indicated that they thought a lot about what to eat next. Furthermore, sometimes, it was very difficult to stop eating because they used food to control some feelings. They used phrases like “once I open it [a package or a box of some food] I cannot stop eating.”

All OB participants stated that they, or someone in their family, had some kind of health condition, such as hypertension or diabetes, which influenced the way in which they ate. These health conditions influenced what kinds of foods they ate, or how they prepared certain foods. In addition, they reported adapting their diet to their husband’s if he had hypertension, by reducing the amount of salt in all of their meals.

##### Unhappy with the Body

Almost all of the participants in the OB group were dissatisfied with their body image. They said that they did not like how they looked and how this made them feel emotionally. They frequently mentioned how their bodies made it difficult to buy clothes they wanted to wear, and they also complained about how they looked in certain kinds of clothes. As an OB participant said, “You see other girls and they are skinny… all clothes look good on them… and you [referring to herself] go to the store and nothing fits you…You try to flirt with your husband and wear something tighter… so that you can feel and look more beautiful… but, as you are fat, you feel like you are not attractive to him…” (C13-OB).

As they felt bad about their bodies, they constantly changed the way that they ate and tried to go on a diet for short periods of time. In fact, a significant number of OB women indicated that there had been several times in which they had modified their eating habits so as to lose weight; however, they said that as in many of these attempts they had failed to reach satisfying outcomes, they felt frustrated and had given up on trying to eat healthier. Finally, some OB women also stated that these changes were attempted without seeking advice or assistance from a health care provider.

##### Gender Roles

Most OB participants referred to topics related to their role as mothers and wives. They often pointed out that their role in the household was to be the one who had to cook or serve others, especially partners and children. As a result, they were always worried about having dinner ready to serve when their husbands arrived home from work or having lunch ready for their children when they got home from school. As an OB participant pointed out, “If he [her husband] arrives at 3 p.m., I have the table set at 2:45 p.m., his plate in the microwave ready to heat up, the [glass of] juice with ice. if it doesn’t have ice, he does not drink it…” (S12-OB).

They felt that these activities were their duties because of their role as housewives. According to participants, this role was also something that had been passed on to them (and will continue to be passed on) from generation to generation among women in Chilean families.

#### 3.2.2. Themes Found in the OB and Overweight (OW) Groups

##### Perceptions of Difficulties and Barriers

Both groups indicated that eating healthily meant overcoming several obstacles. First, they perceived that during the periods in which they were “dieting,” they got bored because they thought that they were engaging in the same routine every day, or as they said, eating “just lettuce” every day. In contrast, on the basis of how the media presents it, they perceived unhealthy foods as more fun and tempting. An OW participant mentioned, “I wanted to lose weight, so I followed it [the dietary plan] as it was planned… but after a while I got bored, and as the girl [the nutritionist] was not there, I could not ask her about what I could change” (C27-OW). Second, they perceived a healthy diet as more expensive. They pointed out that with a tight budget, eating healthily was very difficult. In addition, they perceived that eating hot dogs, fries, hamburgers, or similar foods, was enough to satisfy the whole family as opposed to giving them healthy food. Another important obstacle to being able to change their diets was the lack of social support from their husbands/partners and children. They said they were unable to cook healthy meals on a regular basis because their families would not like them, or that they preferred to avoid healthy meals altogether due to their cost. For instance, a participant indicated, “I want to make a habit of eating [healthier], but the schedules are difficult… the girls [her daughters] are sometimes hungry and they tell me to make something [to eat], and they make me eat…” (L16-OW). Finally, participants recognized that they had some eating behaviors that were difficult to change and that adopting a new eating behavior that deviated from their usual routine was difficult for them.

#### 3.2.3. Themes Found in the Normal Weight (NW) Group only

##### Not Having Enough Time

**Figure 3:**
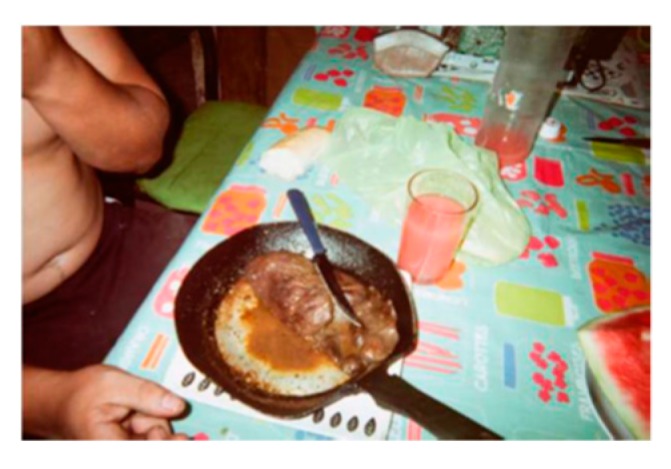
A piece of fried meat.

Most participants in this group indicated that they had to do several different activities at home during the day, such as cleaning the house, taking care of their younger children, taking the older ones to school, etc. Therefore, they said they did not have time for preparing more “elaborate food.” Due to this, they cooked food like pasta, rice, and sausages or what they called “easy and quick-to-prepare food.” In addition to this, they said that as they did not have enough time, they sometimes ate while doing other activities, or they ate really fast. A participant in this group pointed out, “When I am running out of time, I eat this (Figure 3) … Can you see what is it? A piece of fried meat with a glass of juice and bread… I have so many things to do during that day… this (Image) was my lunch; I did not have time to cook [something else]” (V22-NW).

## 4. Discussion

This study discusses the similarities and differences between those elements that influence women’s diets according to their weight status: normal weight, overweight, and obese. All participants in our study had similar (low) socioeconomic backgrounds (Table 1), which allowed us to contextualize the findings in a more specific manner and also explore different influences on eating habits without the interference of major socioeconomic variables. To our knowledge, just one previous study has examined food choices in low SES women of different weight statuses in the USA [36], indicating that several factors would be influencing eating behaviors and the current weight status in women. The current research advances our knowledge by providing information that explores in-depth factors that influence what vulnerable women choose to eat according to their weight status. The present study offers additional, non-biological information that can be used by health care providers and health planners to improve women’s diets by promoting healthy eating in both healthy women and overweight women.

We used qualitative and quantitative methods to compare the data for each weight group. The qualitative analyses allowed us to identify differences between the participant’s discourses that may not have been discovered through quantitative analysis alone. Furthermore, when subtle quantitative differences were found between groups, these tendencies seldom achieved statistical significance due to insufficient statistical power. Employing both types of analysis helps to enrich the interpretation of a studied phenomenon [49]. Hanna and Lautsch recommend employing similar approaches because they help to “build on other findings and add to them, enabling researchers to develop new insights into their phenomena of interest” [50] (p. 16).

In trying to understand women’s eating behaviors, we noticed that food decisions of participants who have obesity are more complex because those decisions include multiple factors. Among the four factors that the three weight status groups had in common, overweight or obese participants had more to say about them. Those two groups tended to repeat those factors several times in their discourses (even when this was not statistically different), and more patterns were found in these groups as well. Furthermore, in the analysis per weight status group, most of the new factors were found in participants with obesity. This finding could explain why it is so difficult for women who are obese or overweight to change their eating behaviors as their diet is determined by multilevel factors that are not always under their control (e.g., family).

Among the themes that appeared in the three groups, we found that participants who were overweight or obese tended to talk more about family and financial issues. Both groups tended to justify their behaviors based on their family more often than the normal weight group, which suggests that those two groups may be more dependent on others when deciding their own diets. It seems then that obese and overweight women perceive a lack of power to make decisions about their own diets; and most importantly, that they also lack support from their families, which is a trend found in research conducted on women who are overweight [51,52]. Considering family dynamics and the support of key family members may be crucial to improve the efficacy of programs aimed at promoting healthy weight among women from low socio-economic groups. In addition, participants with obesity tended to repeat certain topics more often, such as their lack of money, other financial problems, and how this poses a problem for eating healthier. The women’s repeated reference to the lack of enough money (a tendency found more often in women with obesity) could be explained by the fact that they were from poor neighborhoods where food insecurity has been well-documented [53,54]. Similar findings have been found by Dressler and Smith [36] who state that women who are overweight or obese talked more about food prices, costs, and other financial issues than women with normal weight.

In the group analyses, we found new factors that determined women’s diets, mostly in the obese group. Poor mental health and poor physical health (their own or that of a family member), problems with their appearance, past experiences of failure, gender issues, and barriers to eating healthily add more factors to the complex “food world” in which women with obesity find themselves. These findings are in line with previous research that has studied people with obesity. They tended to have more symptoms of depression [55], more body dissatisfaction [56], more negative attitudes toward their weight [57], lower rates of success in previous attempts to reduce weight, or several barriers for eating healthier [52]. These factors may lead to higher rates of attrition in obesity treatment programs [58]. Furthermore, in our study, participants with obesity demonstrated that their role as mothers and wives was strongly associated with their diets, demonstrating an effect of their gender role consistent with previous research [59,60]. In addition, women with overweight or obesity perceived more barriers to healthy eating. Becoming bored with dieting was mentioned by several of these participants. This could be explained by the fact that people with fewer years of education and lower income may have less variety in their food selections [61,62,63]. Perceptions of healthy food being expensive, lack of social support, being accustomed to eating in certain way, were other reasons mentioned by the women with overweight and obesity to explain their eating behaviors.

Our findings suggest that working with obese women could present a greater challenge than working with women with other weight statuses. It is in this group that more psychological factors have been found. This could be related to the emotions observed in obese women or the use of food as a coping strategy for their problems and stress [36,64]. Consequently, in comparison with the other groups, they may be more prone to select unhealthy food as seen in previous research [65]. Added to this, environmental factors such as family preferences and the cost of food seemed to have stronger effects among obese women than for the other groups.

Despite its contributions, this study has some limitations. First, the three groups each had a small sample size, particularly the obese group which impacted the statistical analyses. In addition, in the qualitative analyses, data saturation may not have been achieved for the OB group. Second, weight was measured at different times during the day depending on when participants were available (midday, afternoon, or evening). This could have led to small differences across subjects with respect to the stability of the weight data. The women in our normal weight group were found to have a mean BMI closer to the upper level of that category for women living in Chile. This could be a function of the current trend in Chile and other parts of the world in which BMI values are progressively increasing for all weight status groups [66]. Third, all participants had a partner and children (inclusion criteria), which could explain why the influence of family members appeared frequently in the photographs and narratives. It would be interesting for future research to explore if the eating behaviors of single women are also influenced strongly by their family member. Fourth, the participants’ decision about what to photograph may have been biased by the fact that this was a study of food and nutrition. Finally, the frequency of the temporality theme may have been influenced by the fact that this research was conducted during the summer, when people can find a greater variety of fruits and vegetables.

The present study investigated the determinants of women’s diets according to their weight status, using quantitative and qualitative analysis from photo-elicitation interviews. Factors related to family, temporality, financial issues, and perceptions about food were found to hold true across all weight statuses. Overweight or obese women showed stronger patterns within these factors, with family often playing the most important role. Three other themes were found mostly among women with obesity: these women were more likely to report depressed affect and anxiety as explanations for why they eat unhealthy food. Also, they were more likely to feel unhappy with their body which could generate more anxiety and stimulate overeating. Women with obesity often reported that they were more worried about their family’s health rather than their own, disregarding their own diet in favor of their family preferences. Those who were overweight or obese also perceived more barriers to eating healthier.

Of the three groups studied, women with obesity seem to have a more multilayered “food world.” The “food world” represents everything in a woman’s life that relates to food and that is important for her. Our findings suggest that women with obesity base their food choice decisions on individual and environmental factors that are difficult to control by themselves. This could imply that it is more difficult for them to make healthy food choices and, therefore, more difficult to change unhealthy eating behaviors because these women perceive complex barriers to do so. Consequently, additional research on socio-cultural and contextual determinants of eating behavior is needed in order to develop more culturally-competent approaches that adjust to the “food world” of women with obesity.

Our research supports the conclusion that in order to treat obesity effectively, it is necessary to take into consideration intrapersonal factors that influence women’s self-efficacy, self-control, and self-esteem. Furthermore, it is critical to ponder factors from the individual’s environment in the development and implementation of obesity treatment programs for this population. The development of nutritional guidelines for this population will require health care providers and health policy makers to pay close attention to these socio-cultural and contextual factors.

## Figures and Tables

**Table 1:** General characteristics of participants by weight status (*n* = 31).

Characteristic	Normal Weight *n* = 9	Overweight *n* = 15	Obese *n* = 7	*P*
*Age in years, mean* ± SD	36 ± 5.9	35.1 ± 5.4	38.8 ± 8.6	0.65
*Number of children, median (IQR^1^)*	2 (2)	2 (1)	3 (2)	0.44
*Educational attainment* n (%)	0.16
≤12th grade	1 (11.1)	0	2 (28.6)
High School	4 (44.4)	12 (80)	4 (57.1)
Technical	33.3 (3)	20 (3)	14.3 (1)
College	11.1(1)	0	0
*Work status*	1
Unemployed	6 (66.7)	9 (60)	4 (57.1)
Employed	3 (33.3)	6 (40)	3 (42.9)
*Household income US$ per capita/month, median (IQR)*	154.2 (256.2)	144 (114.9)	137 (49.6)	0.90
*Kind of health insurance* n (%)	0.71
Public	100 (9)	93.3 (14)	85.7 (6)
Private	0	6.7 (1)	14.3 (1)
*Presence of NCD*^2^ n (%)	0.11
Yes	7 (77.8)	11 (73.3)	2 (28.6)
No	2 (22.2)	4 (26.7)	5 (71.4)
*Any kind of aerobic exercise* n (%)	0.15
Yes	4 (44.4)	4 (26.7)	0
No	5 (55.6)	11 (73.3)	7 (100)
*Any kind of resistance exercise* n (%)	0.52
Yes	1 (11.1)	0	0
No	8 (88.9)	15 (100)	7 (100)
*BMI^3^ kg/m^2^*, *median (IQR)*	23.9 (1.7)	27.8 (3.5)	35.2 (7.6)	<0.001
*Calorie intake* (*n* = 22), *mean* ± SD	2213.4 (1051)	2403.80 (977)	2190 (618)	0.88

1. Interquartile range. 2. Non-communicable diseases. 3. Body mass index (Kg/m^2^).

**Table 2:** Summary of the patterns within themes by weight status group.

Theme-Variable/Weight Status	Normal Weight	Overweight	Obese
Family	I cook it because others asked me for it I eat it because others told me about it	I cook it because others asked for it I cook it because others like it I ate it because it represents a family moment I eat it because they brought it to me	I cook it because others asked for it I cook it because others like it I ate it because it represents a family moment I cook it because it is good for them I rely on what others decide
Temporality	I eat special food during the weekend My food intake depends on the weather During this season there are special foods	I eat special food during the weekend My weekends are with family My food intake depends on the weather During this season there are special foods My fruit and vegetable intake changes during the year	I eat special food during the weekend My weekends are with family My food intake depends on the weather During this season there are special foods My fruit and vegetable intake changes during the year
Financial issues	I control the money… I cannot eat it because I do not have money… I always look for the cheaper one…	I control the money… I cannot eat it because I do not have money… The money that I have is not enough I eat that food when I have money	I control the money… I always look for the cheaper one… The money that I have is not enough This food is so expensive I would eat healthier if I had more money
Perceptions about food	This food is good/bad, so I eat/do not eat it… This food has more nutrients or is good/bad for my health	This food is good/bad, so I eat/do not eat it… This food has more nutrients or is good/bad for my health I must have this food This food increases my weight	I must have this food This food increases my weight I have heard some myths about it

The same patterns between the groups have the same colors. The black patterns are those that appeared only in the corresponding group.

**Table 3:** Comparison of percentage of participants who mentioned a theme by participant’s weight status.

	Normal Weight *n* = 9	Overweight *n* = 15	Obese *n* = 7	*p*
Family	100	100	100	n/a
Temporality	100	93.3	85.7	0.71
Financial issues	88.9	93.3	85.7	1
Perceptions about food	88.9	100	100	0.52

n/a = not applied.

**Table 4:** Comparisons between the repetitions ^a^ of themes mentioned by the women according to weight status.

	Normal Weight *n* = 9	Overweight *n* = 15	Obese *n* = 7	*p*
Family	13.7 ± 7.9 ^b^	14.9 ± 8.1 ^b^	17.3 ± 6.5 ^b^	0.65
Temporality	7 (6.5) ^c^	7 (3) ^c^	6 (12) ^c^	0.64
Financial issues	4 (6.5) ^c^	5 (5) ^c^	14 (18) ^c^	0.13
Perceptions about food	7 (3.5) ^c^	4 (5) ^c^	8 (6) ^c^	0.23

a. Total number of times that each theme was mentioned/number of participants. b Mean ± SD. c Median (IQR, interquartile range).

**Table 5:** Other factors that influence women’s eating behaviors by weight status.

Theme/Variable	Normal Weight	Overweight	Obese
Mental and physical health	0	0	“There is something in my health that does not allow me to eat normally…”
Unhappy with the body	0	0	“I do not like how I look…”
Gender role	0	0	“It is what I have to do…”
Perception of difficulties and obstacles	0	“It is so difficult to eat healthier”	“It is so difficult to eat healthier”
Perceptions of lack of time	“I do not have time…”	0	0

0 = absence. In the case of the obese group, presence was considered if the theme was found in 71.4% (5 participants) or more of overall participants.

## Supplementary Materials

The following is available online at https://www.mdpi.com/2072-6643/11/8/1809/s1: Supplementary material Questionnaire: Demographic, socioeconomic and health status form.
