# Supplementary material for: Weight Matters—Factors Influencing Eating Behaviors of Vulnerable Women
Source: Nutrients. 2019 Aug 6;11(8):1809. doi: 10.3390/nu11081809 (PMC6723940; doi:10.3390/nu11081809)
Supplement: Supplementary file 1 [file nutrients-11-01809-s001.pdf]

Demographic, Socioeconomic and Health Status Form

English version

Date of interview:		Participant's Number:
**PARTICIPANT'S CHARACTERISTICS**		
**DEMOGRAPHICS**		
**1. Date of Birth:**		
**2. Marital Status:**	❑ Single<br>❑ Cohabitant<br>❑ Married<br>❑ Divorced<br>❑ Separated<br>❑ Widow	
**3. Children in charge**	❑ Yes<br>❑ No	
	Number of children:	
	Ages of children:	
**4. Educational Attainment**	❑ Elementary<br>❑ Vocational<br>❑ Master / PhD<br>❑ High School<br>❑ Higher Education<br>❑ None	
**5. Level of Employment**	❑ Employed<br>❑ Unemployed	
	Current or past occupation:	
**6. Type of housing**	❑ House<br>❑ Apartment	
	❑ Other:	
**7. Homeownership**	❑ Own<br>❑ Rent	
	❑ Other:	
**8. Number of people that live at home:**		

<table>
<tr><td rowspan="2">9. Head of household</td><td colspan="2">❑ Participant
❑ Spouse / Partner
❑ Parents in law
❑ Parents of the participant
❑ Son / daughter</td></tr>
<tr><td>❑ Other:</td><td></td></tr>
<tr><td rowspan="2">10. People that contribute economically at home</td><td colspan="2">❑ Participant
❑ Parents in law
❑ Spouse / Partner
❑Parents of the participant
❑ Son / daughter</td></tr>
<tr><td>❑ Other:</td><td></td></tr>
<tr><td colspan="3">If the participant is not the head of household, please answer the following questions about the person who is the head of the household:</td></tr>
<tr><td>11. Educational Attainment</td><td colspan="2">❑ Elementary
❑ High School
❑ Vocational
❑ Higher Education
❑ Graduate
❑ None</td></tr>
<tr><td rowspan="2">12. Level of Employment</td><td colspan="2">❑ Employed
❑ Unemployed</td></tr>
<tr><td>Current or past occupation:</td><td></td></tr>
<tr><td colspan="3">13. Total income at home:</td></tr>
</table>

HEALTH RELATED INFORMATION		
**14. Health insurance**	❑ Public<br>❑ Private	
**15. Presence of Chronic Disease**	❑ Yes<br>❑ No	
	❑ Diabetes<br>❑ Hypertension<br>❑ Hypercholesterolemia<br>❑ Hypertriglyceridemia	
	❑ Other:	
PHYSICAL ACTIVITY		
During the past month, other than your regular job, did you participate in any physical activities or exercises such as running, calisthenics, golf, gardening, or walking for exercise?<br><br>❑ Yes<br>❑ No		
17. What type of physical activity or exercise did you spend the most time doing during the past month?		
ANSWER:		
